# A short-term bioreactor assay to assess the effect of essential oils on a microbiota derived from piglet’s intestinal content

**DOI:** 10.1186/s13028-023-00679-w

**Published:** 2023-05-19

**Authors:** Mathieu Bellerose, Philippe Fravalo, Isabelle Mainville, Yves Arcand, Alexandre Thibodeau

**Affiliations:** 1 Research Chair in Meat Safety, Faculty of Veterinary Medicine, University of Montreal, 3 200 rue Sicotte, (J2S 2M2), Saint-Hyacinthe, Canada; 2 Swine and Poultry Infectious Diseases Research Centre, Faculty of Veterinary Medicine, University of Montreal, 3 200 rue Sicotte, (J2S 2M2), Saint-Hyacinthe, Canada; 3 Agriculture and Agri-Food Canada, Saint-Hyacinthe Research and Development Centre, 3 600 Casavant O, Saint-Hyacinthe, J2S 8E3 Canada; 4 Chaire Agro-alimentaire, Conservatoire national des arts et métiers, Le Cnam, 2 Rue Camille Guérin, Ploufragan, 22440 France

**Keywords:** Bioreactor, Essential oils, Microbiota, Piglet

## Abstract

**Background:**

Modulating the microbiota is an emerging way to improve pig health. *In-vitro* bioreactor systems can be used to reproduce intestinal microbiota to study modulating avenues. In this study, a continuous feeding system to support a microbiota derived from piglet colonic contents, over 72 h, was developed. The microbiota from piglets was collected and used as inoculum. The culture media was derived from an artificial digestion of piglet feed. The microbiota diversity in time, the reproducibility between replicates and the diversity of the bioreactor microbiota compared to the inoculum was assessed. Essential oils were used as a proof of concept to assess the *in vitro* microbiota modulation. The microbiota diversity was assessed by 16S rRNA amplicon sequencing. Quantitative PCR was also used for total bacteria, lactobacilli and Enterobacteria.

**Results:**

At the start of the assay, the bioreactor microbiota diversity was similar to the inoculum. Time and replication affected the bioreactor microbiota diversity. Between 48 and 72 h, no statistical variation of the microbiota diversity was observable. After a 48 h running period, thymol and carvacrol were added at 200 ppm or 1000 ppm for 24 h. No microbiota modification was observed by sequencing. Quantitative PCR results showed a significant growth of lactobacilli when thymol was used at 1000 ppm, where only a trend was observed with the 16S analysis.

**Conclusions:**

This study presents a bioreactor assay that can be used as a tool for rapid screening of additives and suggests that the effects of essential oils on the microbiota are subtle, acting against a few bacterial genera.

**Supplementary Information:**

The online version contains supplementary material available at 10.1186/s13028-023-00679-w.

## Background

The intestinal microbiota of the pig is a complex community living in homeostasis with its host [1]. This microbiota is essential to the host, performing important functions such as the modulation of intestinal tissue proliferation and differentiation, stimulation of the host immune system, energy metabolism, vitamin catabolism, and protection against pathogen colonization [2–4]. The composition and structure of the bacterial community found in a pig’s gut is largely determined by factors such as diet, age, genetics, environmental conditions, microbial infection, and antimicrobial exposure [5]. In monogastric mammals, when the equilibrium of the intestinal microbiota is disrupted, a phenomenon called dysbiosis occurs [6]. In an attempt to restore the balance, the microbiota can be modulated by the addition of in-feed probiotics and prebiotics (14), by introducing antibiotic treatments (when pathogens are present), or by drastic clinical interventions such as fecal microbiota transplantation [7, 8].

In pigs, strong diet transitions (e.g. weaning or feed program transitions) are critical stages that could be deleterious on the gut microbiota composition, causing dysbiosis [9]. During this phase, a reduction of *Lactobacillus* and an increase of *Proteobacteriaceae* such as *Escherichia coli* has been reported [10]. Some experimental studies conducted in live pigs described the benefits of different feed additives on the piglet microbiota composition and diversity, during the post-weaning period. Indeed, the addition of an essential oil mixture of thymol and cinnamaldehyde was shown to reduce the fecal *E. coli* number by one log of magnitude, leading to an increase of the lactobacilli to *E. coli* ratio, which is considered a favorable marker for intestinal health [11]. Other *in vivo* studies described that the addition of cello-oligosaccharide in the feed, as a prebiotic, led to an increase in *Lactobacillus* but had no effect on the *E. coli* population. However, other studies reported that the addition of mannan-oligosaccharides in the feed as a prebiotic had no impact on *Lactobacillus* but decreased *Enterobacteriaceae* in the analyzed fecal samples [12, 13].

In pig production, some inhabitants of the intestinal microbiota are foodborne pathogens, and some of them – such as *Salmonella* – can act as intestinal pathogens for the pigs as well, causing diarrhea [14, 15]. Therefore, the pig intestinal microbiota plays a crucial role in both pig and public health, making it a prime target for intervention. However, data regarding the mode of action of microbiota modulation options remain limited with controversial results between studies, creating an urgent need for more research on the topic.

To study animal microbiota, *in vivo* models are conducted in animal facilities under controlled conditions or are conducted on farms [16]. However, using animal models is costly, time consuming and may raise ethical concerns. Since the microbiota is modulated by multiple factors in animal models, perfectly controlling only one or a few environmental variables at a time can become a tedious task, especially in field conditions. In an *in vitro* approach, culture conditions can be meticulously controlled so that a single variable or an individual bacterial genus can be investigated over a precise period, provided that the targeted bacteria can be cultured. Reproducing complex interactions between the numerous bacterial genera or strains for longer time requires a methodological approach more complex than standard culture. Co-culture conditions need to be adequate for growth of all bacterial candidates, culture media needs to be replenished periodically if a longer culture time is needed, and sometimes the study context calls for specific yet stable environmental conditions to relate to a given environment like the gut [17]. To minimize these limitations, bioreactor systems can be employed for *in vitro* studies.

Bioreactor systems used for microbiota studies vary in complexity. The simplest model is the batch system. In this system, a specific working volume is set, and no additional nutrients are added throughout the experiment [18, 19]. These systems are designed for short-term experiments. A more complex system is chemostats. This system involves the addition and removal of culture media, keeping nutrient availability and bacterial waste stable for weeks [20, 21]. Based on this system, other components can be added to reproduce or simulate specific aspects of the host microbiota [22, 23].

Bioreactors were also used for studies on the animal microbiota using the pig as a model. For example, a continuous bioreactor by Tanner et al., was used to stabilize a pig microbiota that could be distributed in five parallel reactors [23]. Some bioreactors systems require a long stabilization period, consisting of over a week to months [23, 24], which reduces the number of effective experiments that can be done as well as increases the chance of possible system crash due to a bioreactor malfunction. Also, some of these systems rely on a single reactor, thus limiting the number of conditions tested simultaneously [24, 25]. Finally, most systems use multi-ingredients lab-made culture media for the bioreactors that could induce some unwanted change in the microbiota composition compared to *in vivo* conditions [23, 24].

The aim of the present study was therefore to use a bioreactor system that can maintain a microbiota derived from a piglet’s intestine for the fast screening of feed additives intended to modulate the intestinal microbiota. The first objective was to assess if this system could be used – over the period of 72 h – to enable the eventual high-speed screening of some microbiota modulation options as a way to rapidly select the best-performing feed additives for use in future *in vivo* studies. The second objective was to, as a proof of concept, use essential oils to assess if the bioreactor microbiota can be modified. To achieve these objectives, high throughput sequencing of the genes coding for the 16S rRNA of the bioreactor’s microbiota, at key time points, was used and some results confirmed by qPCRs. Firstly, the microbiota evolution over time was characterized. Secondly, the reproducibility of the assays was tested by comparing results between 3 different replicates. Thirdly, the bioreactor microbiota was compared to the inoculum microbiota as well as the microbiota of live piglets sampled in another study. Lastly, the impact of the addition of essential oils on the bioreactor microbiota was assessed.

## Methods

### Bioreactor system

The bioreactor system was composed of eight custom-made jacketed glass reactors (Soham Scientific, Fordham, UK). The mother reactor had a maximum volume of 600 mL and was connected to seven 300 mL daughter reactors (Additional File 3). These reactors were each placed on an individual magnetic stirrer (Fisher Scientific, Ottawa, ON, Canada) and set at speed 3, the lowest speed that allowed the full steering of the culture media, to keep the microbiota from sedimenting. A water circulator (Polyscience, Niles, Il, USA) was connected to the glass jacket of the reactor, enabling the temperature to be maintained at 39 °C [26].

Each bioreactor had a pH regulating system composed of a pH probe (Hach, London, Canada) connected to a custom-made control panel (Les Contrôles Luc Hébert Inc, Saint-Hyacinthe Canada) that activated, when needed, a set of two pumps (Kamoer, Shanghai, China) – one for acid (HCl, 1 M) and one for base (NaOH, 2.5 M). Software was used to set a pH profile for each reactor, which could vary in time if needed. Before every experiment, pH probes were carefully calibrated with standardized solution at pH 4, 7 and 10. Once each experiment was over, the software generated an hourly report of the pH and the use of all solutions throughout the experiment.

Gaseous nitrogen was constantly injected into each reactor at 150 kPa, the lowest possible pressure, to remove gaseous oxygen. A set of calibrated pumps (Cole-Parmer, Montreal, Canada) was used to constantly add fresh culture media to the reactors, at a rate of 16 mL/h, the rate was chosen based on another *in vitro* model, the Polyferm, method which used a nutritive medium at 26 ml/h [23]. One pump was used for feeding the mother reactor and another pump with a multichannel head was used for feeding the daughter reactors.

Culture media was thawed overnight every 24 h and, until added to the bioreactors, kept refrigerated at 4 °C on a plate stirrer (Fisher Scientific, Ottawa, Canada) to prevent the media from sedimenting (described in detail in the next section). Culture media was added to the reactors at room temperature. Another set of pumps (Cole-Parmer, Montreal, Canada) with multichannel heads was used to remove the volume excess from the daughter reactors. This excess of media was collected in closed 5 L glass bottles with four-hole caps (Diba Industries, Danbury, CT, USA) for tubing, therefore allowing the safe recovery and decontamination of all effluents.

The cap of each reactor was composed of three GL-25 connectors and three 7 mm connectors. The GL-25 connectors were used for the following: one for the pH sensor; one divided into four connections to introduce the acid, the base, and the nitrogen into the culture media and to release excess gas in the reactor into an Erlenmeyer containing water (a gas trap to preserve anaerobic conditions); and the last one remained unused and was sealed off. For the three 7 mm connectors, one was for feeding the reactor, one for removing excess volume, and the last one for sampling the microbiota.

### Upper *in vitro* digestion and culture media preparation

Culture media used in this system came from an *in-vitro* digestion of piglet feed. Feed and spring water were fed to a piglet version of the IViDiS system, that could simulate the digestion of the equivalent of 10 piglets in one digestion, in order to provide enough digestate (culture media) for the following experiments. The IViDiS is a dynamic *in vitro* model that attempts to mimic the digestive system of monogastric mammals [27, 28], in this study a piglet, from the mouth to the ileum. Chemicals purchased for Sigma Aldrich (Oakville, ON, Canada) were as followed: Pepsin (P7000; EC Number 232-629-3), pancreatin (P1750, EC Number 232-468-9), α-amylase (A3176) and mucin from porcine stomach (M1778). Lipase was purchased from Bio-Cat Inc (Troy, VA, USA). Porcine bile extract (CAS 8008-63-7) was purchased from Santa Cruz Biotechnology (Dallas, TX, USA). Brush border membrane extract (BBME), isolated from healthy piglets obtained from another study, was prepared in-house using the method described by Cheeseman et al. [29] Enzymes activities were measured according to the protocols proposed by the INFOGEST international consortium [30, 31], except for peptidase activity in BBME which was measured according to the method described by Pfleiderer [32]. Physiologically relevant solutions were used to control the pH profiles in both the stomach and duodenum reactors.

Piglet feed was first ground and sifted through a 1 mm sieve. For digestion, 1.5 kg of sieved feed was added gradually into the stomach reactor with 4.5 L of water (Eska, St-Mathieu-d’Harricana, Canada). A digestion profile was developed from *in vivo* data and a review of *in vivo* data cited in the scientific literature to represent the piglet digestive system. The *in vitro* ileal digestate was previously compared to *in vivo* ileal digestates from piglets, using SEC-HPLC to validate protein/peptide profiles and sugar profiles. Protein/peptide profiles were very similar between the *in vitro* ileal digestate and the *in vivo* samples (data not shown). Carbohydrates available in the *in vitro* digestate were higher than in the *in vivo* samples, since the IViDiS model does not simulate absorption. Dilution studies were performed to determine the correct ratio of digestate to diluent to be used. Digestate was collected in sterile plastic bags (Whirl-pak, Madison, USA) immersed in iced water. Bags were changed every 30 min, during the digestion period and frozen at -20 °C until use. Digestate appearance varied in the bags based on the digestion time, with larger particles of undigested feed being present at the beginning (around 6 h after the beginning of digestion) and less at the end (around 9 h after the beginning of the digestion). Instead of mixing all the bags together it was decided to not use the first and last three bags of each digestion, and simply used the bags that showed similar consistency, in order to have batch to batch stability.

Before being used in the bioreactor, different fractions representing the whole digestion process were thawed overnight at 4 °C, pooled, and further diluted 1:1 with phosphate buffer saline (Oxoid, Nepean, ON, Canada) supplemented or not with 1% thioglycolate (Sigma-Aldrich, Oakville, Canada). The culture media was mixed using a stomacher for 60 s and a filter bag (Labplas, Sainte-Julie, Canada) was used to remove larger particles of undigested feed (0.33 mm) and thereby prevent the tubing from getting clogged.

### Inoculum preparation

All animal manipulations were approved by the ethics committee of the Faculty of Veterinary Medicine of the Université de Montréal, certificate number 19-Rech-2047. Twelve piglets (3 weeks old, post-weaning period) were kept for one week for acclimation purposes at the Faculty of Veterinary Medicine of Université de Montréal, in Saint-Hyacinthe, Québec, under strict biosecurity conditions. Animals were offered water *ad-libitum* and a standard commercial antibiotic-free feed, the same used for the preparation of the bioreactor culture media. At the end of the one-week period, no clinical signs of any disease were observed so the animals were euthanized and necropsies were performed to collect colonic contents. Mid-colonic sections were isolated and extremities were tied before cutting. These were sent to the lab on ice for further handling. At the lab, the colonic contents were pooled. An equivalent volume of our in-house freezing media (Brucella broth (2.8%), agar (0.12%), glycerol (20%), sucrose (5%), ascorbic acid (0.4%), and powdered milk (5%)), supplemented with 1% thioglycolate (Sigma-Aldrich, Oakville, Canada), was then added. Each pool was aliquoted into tubes of 10 g and kept at − 80 °C for the subsequent inoculation of the bioreactor. An aliquot of 500 mg was collected, flash frozen in liquid nitrogen, and kept at − 80 °C for sequencing purposes. Overall, the necropsy and the lab work took approximately 6 h. The necropsy allowed for the sterile recovery of the future inoculum. Ileal and caecal content were also collected for further studies. Considering that approximately 100 g of intestinal content can be recuperated from the necropsied pigs and that an initial 10 g of intestinal content (used in the mother reactor as inoculum) can be transformed into 8 bioreactor replicates, the 12 necropsied animals have the potential to allow the use of 80 bioreactors. As the microbiota of fecal samples are slightly but different from colonic or caecal samples, it was decided to use, as proof of concept, for inoculum, fresh intestinal content recovered from necropsied animals to be as close as possible to what is actually present in a pig intestine. The system could also easily be adapted to use excreted fecal matter recovered from high health status pigs to further reduce animal use.

### Bioreactor experiments

To start the system, 10 mL of digestate was inoculated with 10 g of pooled piglet colonic matter in the mother reactor while N_2_ was injected in the system. The mother reactor was then filled with digestate containing 0.1% of thioglycolate, for 6 h, at a rate of 80 mL/h for a total volume of 490 mL at 39 °C. During that time, the pH was monitored and maintained at 6.5 [33]. When completed, 70 mL of the mother reactor content was added to each of the daughter reactors. Volume transfers were done by pulling 35 mL with a 60 mL syringe twice (Terumo, Vaughan, Canada). Daughter reactors were then activated for 18 h with constant feeding (using the same digestate preparation), and culture media was removed at a rate of 16 mL/h. After this period, the culture media was changed to digestate without thioglycolate for an additional 48 h and added/removed with a continuous flow of 16 mL/h. To monitor the reactors, samples (5 ml) were taken with a syringe and added to a 15 mL conical tubes (Sarstedt, Newton, MA, USA) just before the filling procedure was started (T0), taken before dividing the mother reactor (6 h after the start (T6)), and taken at the following intervals: 24 h (T24), 48 h (T48), 51 h (T51), 54 h (T54), 60 h (T60), and finally 72 h after the beginning of the experiments (T72). For the essential oil experiments, the same sampling time points were used. The essential oils were added or not right after the T48 sampling. For the essential oil assay, the oils were added directly into the culture media when needed. Thymol (Jefo, Saint-Hyacinthe, Canada) or carvacrol (Beauchamp international, Brossard, Canada) were tested at a final concentration of 200 ppm and 1000 ppm. Each bioreactor sample was centrifuged immediately at 5000 rpm (Sorvall Legend XTR, Thermo Fisher scientific, Waltham, MA, USA) for 15 min, the supernatant was discarded, and the pellet was frozen at − 80 °C until DNA extractions. The experiment was conducted three times using these conditions to obtain independent replicates.

### DNA extraction and 16S rRNA gene sequencing

From each sample, DNA was extracted for sequencing and qPCR using the DNeasy PowerLyzer PowerSoil kit (QIAGEN Inc., Toronto, Canada) following the manufacturer recommendations. DNA was quantified using a QFX Fluorometer (DeNovix Inc., Wilmington, DE, USA) with the QUBIT BR Assay kit (Invitrogen™, Thermo Fisher scientific, Waltham, MA, USA) and normalized at 5 ng/µL with PCR-grade water. High throughput sequencing was then used for the analysis of the microbiota.

The V4 region of the genes coding for the 16S rRNA were first amplified in a Mastercycler ®Nexus (Eppendorf AG, Hamburg, Germany) with a total of 12.5 ng of genomic DNA from each sample and Platinum Superfi DNA Polymerase (Invitrogen, Burlington, ON, Canada). PCR conditions were as follows: 5 min denaturation at 98 °C, followed by 23 cycles of 98 °C for 30 s, 55 °C for 30 s, 72 °C for 3 min, with a final elongation at 72 °C for 10 min. Amplicon quality was verified on an agarose gel containing SYBR Safe DNA gel stain (Invitrogen, Burlington, ON, Canada) and was sent for Illumina MiSeq (250PE) sequencing at Genome Québec. A positive control – ZymoBIOMICS Microbial Community DNA Standard (Zymo Research, Irvine, CA, USA) – and a negative PCR control (use of PCR grade water instead of DNA) were used for quality assessment purposes.

### Sequence analysis and diversity determination

Raw sequencing reads were demultiplexed, quality-filtered, and analyzed using Mothur software [34] version 1.43.0, according to Mothur MiSeq SOP, with the following modifications: the method for pre-cluster was Deblur and the method for cluster was Unique [35], therefore ASV (Amplicon sequence variants) and not OTU were used. Mothur SOP was last accessed on April 27, 2021. Taxonomic assignation of the ASV was done using the Silva 132 Mothur-formatted database. The resulting files were further analysed in RStudio 1.3.1073 using R version 4.0.3. The shared and taxonomy files produced by Mothur were first imported into R. After inspection of raw data, controls were removed and remaining samples were rarefied to the lowest number of sequences within a sample prior to alpha and beta diversity analysis. Observed, Shannon, and Inverted Simpson indexes were used for the alpha diversity analysis. A Kruskal-Wallis statistical test was used to assess significant differences between relevant conditions for the three indexes. The microbiota structure was assessed using both Jaccard and Bray-Curtis dissimilarity indexes and results were visualized with a non-metric multidimensional scaling (NMDS) graph, followed by statistical confirmation using ADONIS (Vegan package) [36] and Pair-Wise ADONIS (pairwiseAdonis) [37] to test differences between groups. Venn diagrams were generated to report either phylum, family, genus, or ASV present only in specific conditions. Prior to the Venn diagrams, sequences present only once across all samples were removed. A Multivariate Analysis by Linear Models (MaAsLin2) using default options [38] was performed to identify specific biomarkers associated with tested conditions.

### Real-time PCR

Real-time PCR was used to confirm 16  rRNA sequencing results. DNA was used for qPCR in a Roche LC96 Real Time PCR (Roche diagnostics, Mannheim, Germany) to estimate the 16 S copy numbers with the primers used for high-throughput sequencing, as well as Enterobacteria and Lactobacilli levels, using primers detailed in Table 1. The 16 S rRNA gene V4 hypervariable region encoding sequences in the genomic DNA for each sample was amplified by PCR using universal primers detailed in Table 1. The qPCR reaction, with a volume of 20 µL, was composed of Evagreen (MBI Evolution, Montreal, Qc; Canada) 1X, 0.3 µM of each primer, and 50 ng of DNA. Reaction was conducted for 5 min at 95 °C followed by 35 cycles of 30 s at 95 °C, 30 s at 55 °C, 180 s at 72 °C, and finished with a high-resolution melting curve. The Lactobacilli qPCR was performed in a volume of 25 µL composed of Evagreen (MBI Evolution, Montreal, Qc; Canada)1X, 0.5 µM of each primer, and 10 ng of DNA. Reaction was conducted for 2 min at 50 °C and 10 minat 95 °C, followed by 35 cycles of 15 s at 95 °C, 60 s at 60 °C, and finished with a high-resolution melting curve. For the Enterobacteria reaction, the qPCR reaction volume was set at 20 µL and was composed of Evagreen (MBI Evolution, Montreal, Qc, Canada)1X, 0.4 µM of each primer, and 50 ng of DNA. Reaction was conducted for 15 min at 95 °C, followed by 35 cycles of 20 s at 95 °C, 60 s at 60 °C, and finished with a high-resolution melting curve. Standard curve used for the Lactobacilli was made from amplicon using the *Lactobacillus acidophilus* ATCC 314 and the standard curve for the Enterobacteria was made from amplicon produced using *E. coli* ATCC 25,922. These amplicons were then quantified, their number of DNA copies was calculated using the calculator from URI Genomics & Sequencing Center [39], and then they were diluted to obtain standards containing 10^8^ to 10^2^ copies per µL. Negative control (PCR grade water instead of DNA) was performed for quality assessment purposes. Total gene copy numbers were thereafter reported as copy number per ng of extracted DNA.

**Table 1:** Primer list for qPCR analysis

Target	Sequence 5’-3’	Reference
16S copies	GTG CCA GCM GCC GCG GTA A	[5]
	GGA CTA CHV GGG TWT CTA AT	
lactobacilli	GCA GCA GTA GGG AAT CTT CCA	[34]
	GCA TTY CAC CGC TAC ACA TG	
Enterobacteria	ATG GCT GTC GTC ACG TCG T	[34]
	CCT ACT TCT TTT GCA ACC CAC TC	

## Results

### Sequencing quality control

For all 109 samples and controls, the total number of sequences, including controls, was 14,447,474 at the beginning of the bioinformatic process. After the cleanup steps done with Mothur, the total number of sequences was 6,954,810. The highest sequence number in a sample was 77,600 sequences, the mean number of sequences per sample was 54,812, and the lowest number of sequences in a sample was 35,434 sequences. Two negative PCR controls contained 149 and 1249 sequences. Two mock community positive controls were sequenced, and they contained respectively 47,094 and 38,455 sequences. The sequencing error assessed with the ZymoBIOMICS Microbial Community DNA Standard (Zymo Research, Irvine, CA, USA) was 0.017%. The mock community relative composition obtained was similar to the manufacturer’s description.

### Microbiota evolution in time

**Fig. 1:**
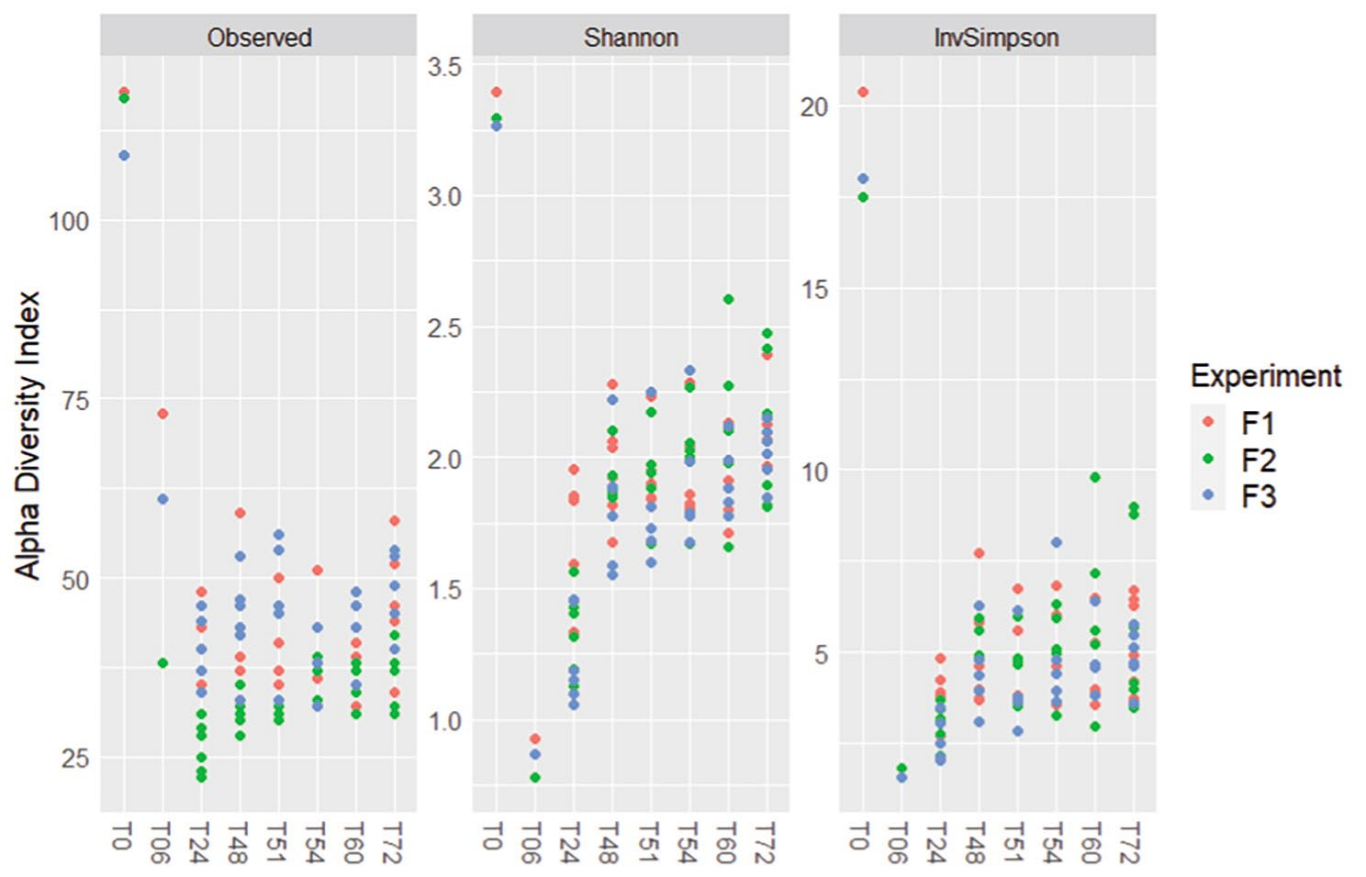
Alpha Diversity of the microbiota according to the time of sampling and the replicate. These graphs represent, respectively, the Observed index, the Shannon index, and the Inverse Simpson index. A significant difference based on time was found for all indexes using the Kruskal-Wallis test (P = 0.004, P = 1.09e-8, P = 2.92e-7). The 3 independent replicates (F1, F2 and F3) are also shown on the graph

The first step was to assess the microbiota diversity throughout the experiments, from T0 to T72. Graphical representations of alpha-diversity indexes for all samples are shown in Fig. 1. A Kruskal-Wallis test was conducted on the three indexes and statistical difference was observed according to sampling time for Observed (P = 0.004), Shannon (P = 1.09e-8), and Inverted Simpson (P = 2.92e-7), where T0 samples presented higher diversity values than all other samples.

Phylum, families, and genus present only at the beginning (T0) or at the end of the experiment (T72) were identified. Additional File 4 shows unique families, their phyla assignation, their total number of sequences at either T0 or T72, and their relative abundance. At the family level, 31 families were unique at T0 and 18 were unique at T72. Sequences from the unique families found at T0 represent 6.1% of all the sequences in the T0 group. The unique families found at T72 represented 0.01% of all sequences recovered at T72.

**Fig. 2:**
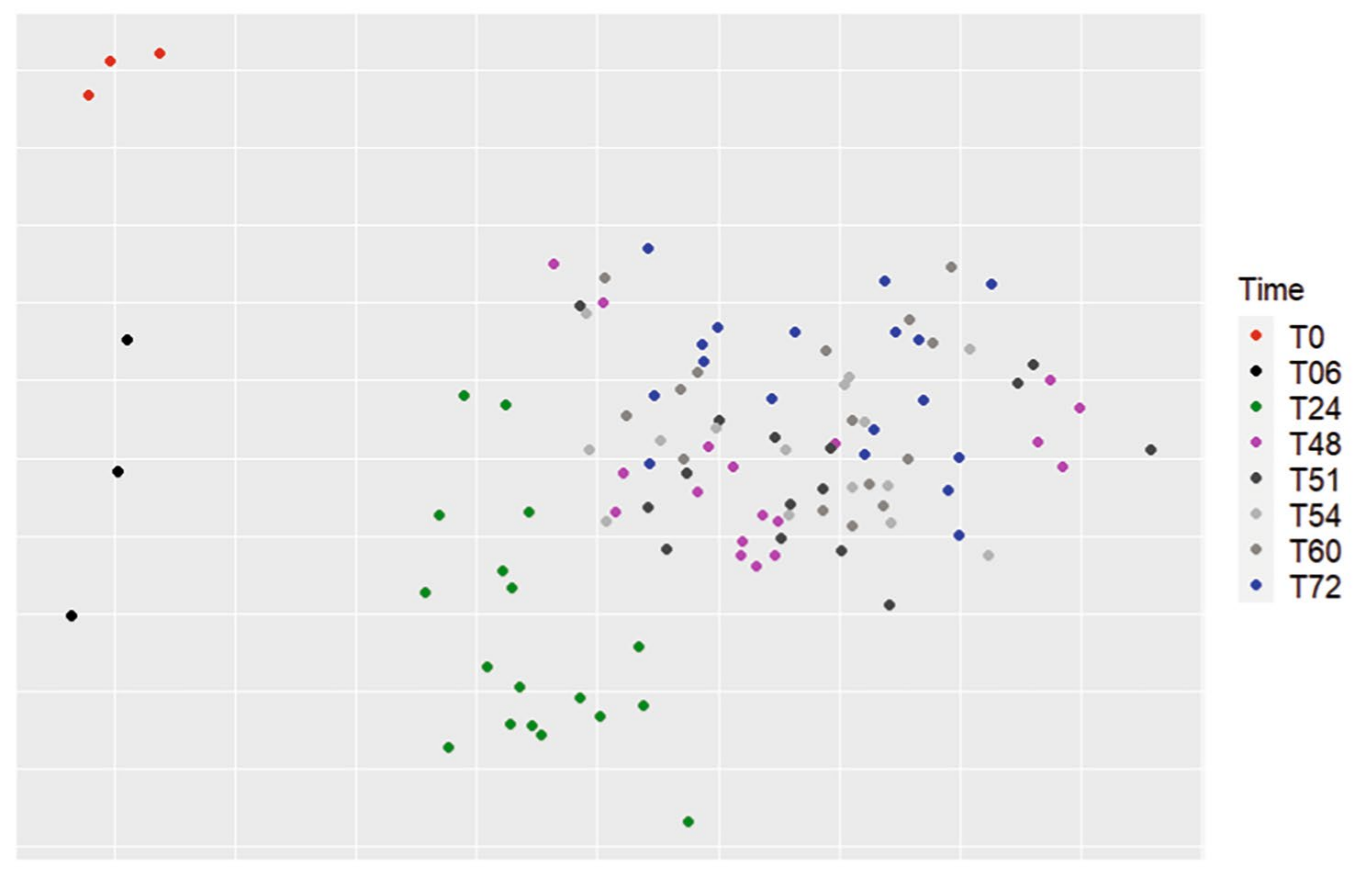
NMDS representation of the beta diversity, using the Bray-Curtis index of the bioreactor samples according to sampling time. A significant difference was found between different times, using the ADONIS statistical test (P = 0.0009). Pairwise Adonis showed a significant difference between T0 and between T24, T48, and T72 (P < 0.01). No statistical difference was shown between T48 and T72

For the beta diversity, the microbiota structure was observed using a non-metric multidimensional scaling (NMDS) (Fig. 2) graphic with the time of sampling as a variable. Using the Bray-Curtis and Jaccard index, differences were observed between sampling times (P = 0.0009). Graphically, samples on the plot clustered according to sampling time, with samples from T48 to T72 occupying the same region on the graph compared to T0, T06 and T24. Pairwise Adonis was conducted to identify significant differences between times: T0 was significantly different from T24, T48, and T72 (P < 0.01) but not significantly different from T06. T06 samples were significantly different from T24, T48, and T72 samples (P < 0.01). No significant difference was observed between samples T48 and T72.

Relative abundance of the microbiota populations at phylum and family levels, at T0, T48, and T72 are shown in a stack bar graph in Additional File 1 and 2. At the phylum level, a clear shift in composition was observed between T0 and T48, and another shift was observed between T48 and T72. At the family level, similar differences could be observed.

Multivariate Analysis by Linear Models (MaAsLin2) was then used to identify bacterial phyla, families, and genera associated with a specific time (using T0 as a reference, N = 3). All MaAsLin2 results are shown in Additional File 4. At the phyla level, eight phyla were less represented at all times compared to T0: Lentisphaerae, Planctomycetes, Verrucomicrobia, Synergistetes, Spirochaetes, Euryarchaeota, Firmicutes, and Bacteria_unclassified. At the family level, 38 families were less represented at all times compared to T0, including Ruminococcaceae, Porphyromonadaceae, and Peptostreptococcaceae. Twelve associations were found for T72, including *Coriobacteriaceae, Leuconostocaceae, Veillonellaceae, Bifidobacteriaceae, and Pseudomonadaceae*.

### Reproducibility of the microbiota constitutions

**Fig. 3:**
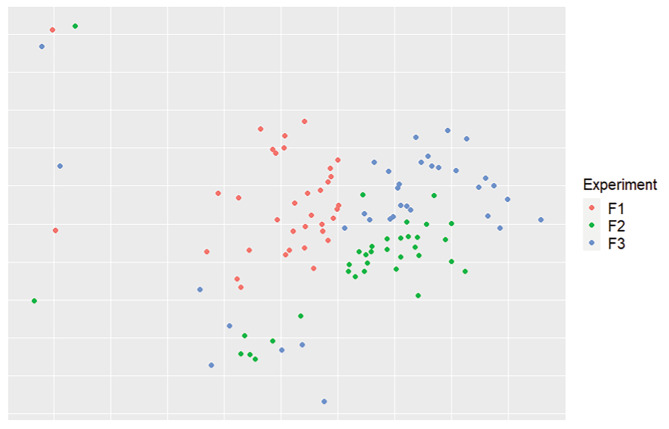
NMDS representation of the beta diversity, using the Bray-Curtis index, based on the experimental replicate. A significant difference was found between different replicate using the ADONIS statistical test (P = 0.0009). Pairwise ADONIS showed a significant difference between all pairings of the experiment (P < 0.001). The 3 independent replicates (F1, F2, and F3) are shown on the graph

The second step was to assess the reproducibility of the experiments, by looking at the alpha (Fig. 1) and beta diversities of the samples using the replicate (F1, F2, or F3) as a variable. For the alpha diversity, only the observed index was statistically significant (P = 8.9e-9) when comparing all samples based on the replicate. For the beta diversity, samples were plotted on an NMDS (Fig. 3), using the replicate as a variable. At first glance, samples from different experiments seemed to regroup according to the experiment. This was confirmed with an ADONIS statistical test using the Bray-Curtis index and the Jaccard index (P = 0.0009). Pairwise ADONIS was conducted to find statistical differences between different experiment pairs. All pairs were significantly different (P < 0.001). When time was added as a variable in the ADONIS test, time, replicate, and the interaction between the 2 variables were found to affect the microbiota structure.

### Similarities between the bioreactor samples, piglet feces, and the inoculum

**Fig. 4:**
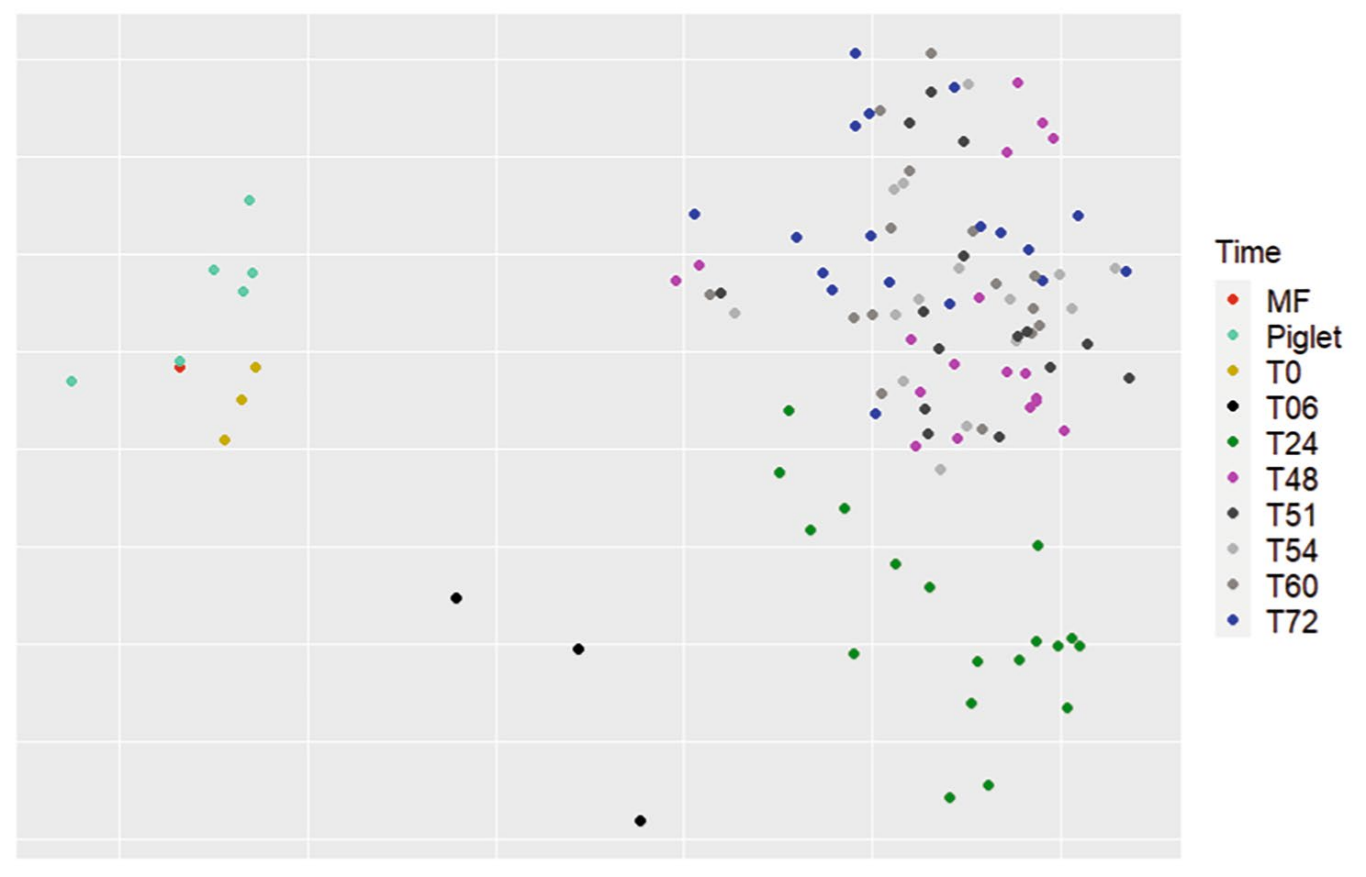
NMDS representation of the bioreactor samples compared to the inoculum and piglet samples, using the Bray-Curtis index, based on time as a factor. A significant difference was found between different times, using the ADONIS statistical test (P = 0.0009). Pairwise ADONIS showed a significant difference between T0 and between T24, T48, and T72 (P < 0.01). Pairwise ADONIS also showed a significant difference between piglet samples and T48, and piglet samples and T72 (P = 0.01). No statistical difference was observed for samples between T48 and T72, nor between piglet samples and MF and T0 samples

The third step was to compare the bioreactor microbiota with the colonic content used for the bioreactor inoculation (MF) as well as with rectal swab samples originating from piglets of another study (healthy negative control animals, 28 days old, raised in a level 2 animal facility) [5]. When comparing the microbiota structures, using the origin of the sample as a variable (MF, piglet, and bioreactor samples), a statistical difference was observed (P = 0.0009). Piglet samples were significantly different from the bioreactor samples at T24 and T72 (P = 0.01) but not from the T0 samples. The same was observed for the inoculum (MF). On the NMDS graph (Fig. 4), samples from piglets, MF, and T0 (start of the bioreactor experiments) were grouped, while the other samples were grouped separately. For the alpha diversities, a significant difference was found between bioreactor samples, piglet, and MF samples for all sampling times and for all three indexes (P = 3.52e-5, P = 2.11e-10, P = 3.89e-9), except for T0 samples that were similar to MF and piglet rectal swabs.

**Fig. 5:**
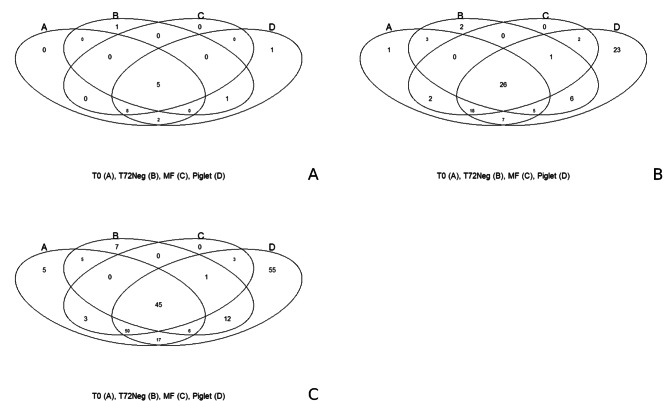
Venn diagram indicating shared and unique phyla (A), families (B), or genera (C) between the sample from the bioreactor at T0, the sample from the bioreactor at T72, the MF sample, and the piglet sample

Venn diagrams were made to represent unique phylum, family, and genus present in the MF sample, the piglet samples, and the T0 or T72 bioreactor samples. The Venn diagrams are shown in Fig. 5. At the phylum level (Fig. 5A), 5 phyla were common between all groups and 8 phyla were found in all groups except the T72. One phylum was unique to T72, and one phylum was unique in the piglet samples. No unique phyla were found in the T0 or MF samples. At the family level (Fig. 5B), two families were found only at T72, 23 families were found only in the piglet samples, and one family was only found at T0. No unique family was found for the MF sample. For the families that were shared, 18 families were common between MF, piglet, and T0, and 26 families were common between all samples. At the genus level (Fig. 5C), seven genera were only found in the T72 samples, 55 genera were only found in the piglet samples, and five genera were only found at T0. Fifty genera were common between MF, piglet, and T0, and 45 genera were common between all samples.

### Effect of essential oils

**Fig. 6:**
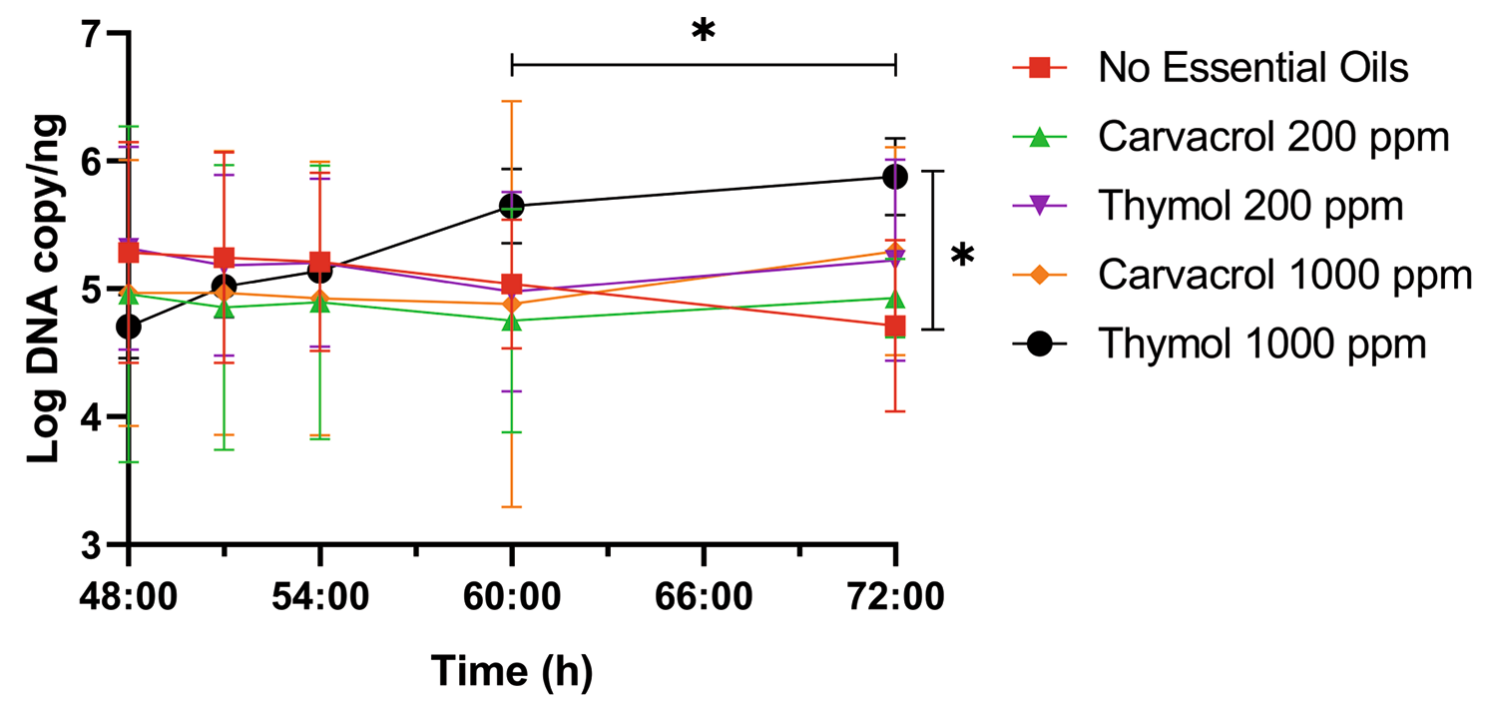
Graphical representation of the evolution of the log DNA copies of lactobacilli per ng of genomic DNA. N = 3, bar represents standard deviation. Lactobacilli levels were measured by qPCR. Graphical representation was generated using GraphPad. Student T-test showed a significant increase in the thymol 1000 samples, between 48 and 72 h of running time (P = 0.001). Another significant increase was found for the thymol 1000 sample compared to the reactor without oil at T72 (P = 0.001)

The next objective was to assess if the bioreactor microbiota could be modified using two essential oils – thymol and carvacrol – at 200 ppm or 1000 ppm, respectively, when added directly in the culture media. Both oils, regardless of the concentration used, had globally no effect on the bioreactor microbiota, the total 16S copy numbers and Enterobacteria levels. However, using qPCR, lactobacilli levels increased in the presence of thymol (but only at 1000 ppm), between the moment the oil was added (T48) and the end of the experiment (T72) (P = 0.001). The same observation could be made at T72 when comparing the experiment with thymol to the control condition (without oil) as shown in Fig. 6.

**Fig. 7:**
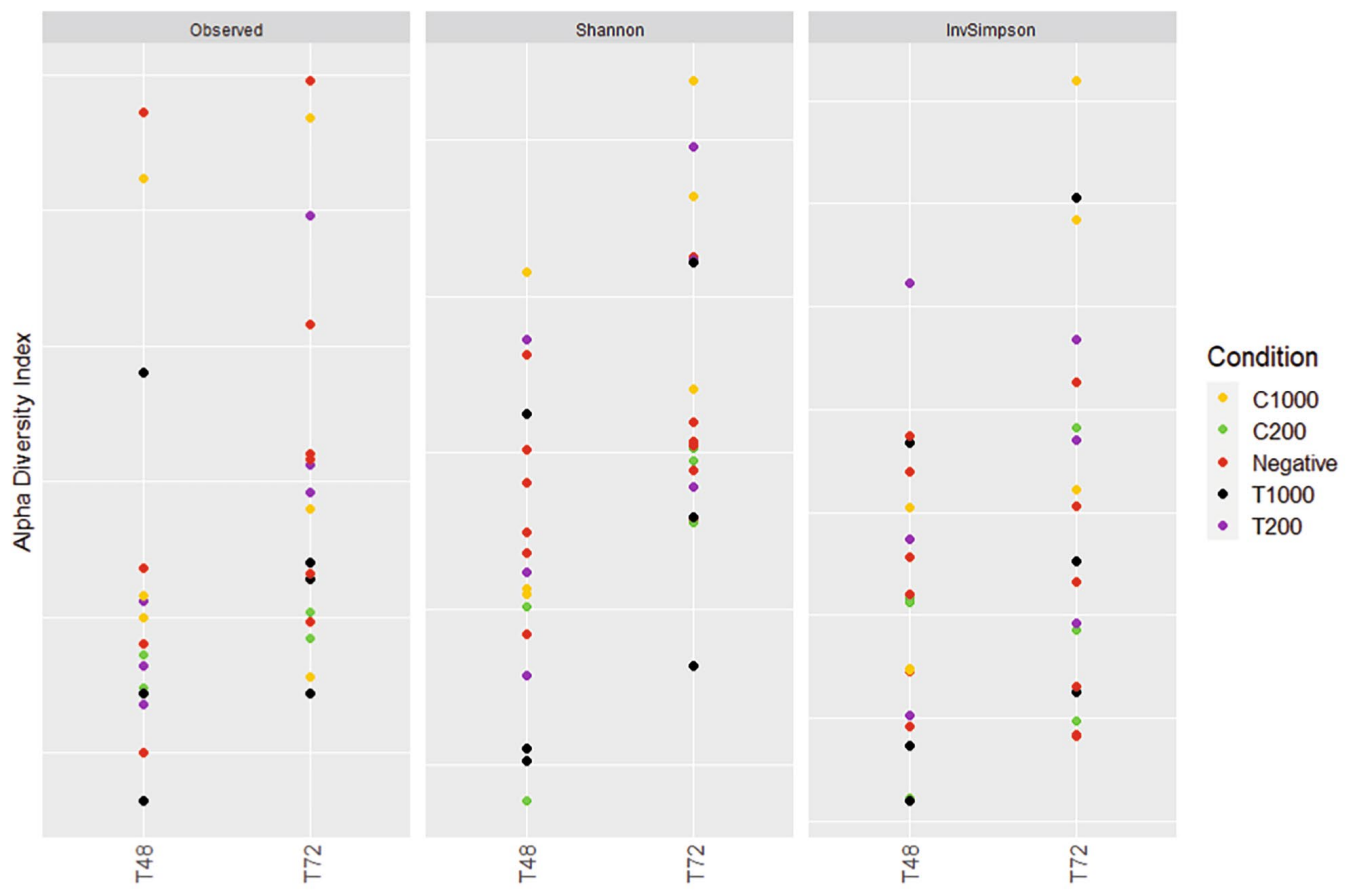
Alpha diversity of the bioreactor samples, between T48 and T72, with condition as a factor. These graphs represent, respectively, the Observed index, the Shannon index, and the Inverse Simpson index. C = carvacrol; T = thymol; negative = control condition without oil addition; 1000 or 200 expresses the final oil concentration in ppm. No significant difference was found between conditions using the Kruskal-Wallis test

**Fig. 8:**
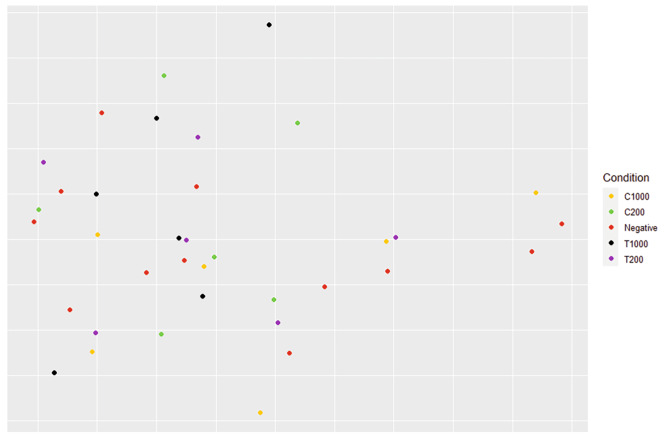
NMDS representation of the bioreactor samples, using the Bray-Curtis index, of T48 and T72, based on condition as the factor. C = carvacrol; T = thymol; negative = control condition without oil addition; 1000 or 200 expresses the final oil concentration in ppm. No significant difference was observed between the different conditions using the ADONIS statistical test

This specific change was not observed at the alpha diversity level (Fig. 7). Using the Kruskal-Wallis test, no significant differences were measured at any time for any conditions. Graphical NMDS representation of the beta diversity is shown in Fig. 8. ADONIS statistical analysis did not reveal any significant difference using the treatments as variables, using both Bray-Curtis and Jaccard indexes.

Considering specific change revealed by qPCR, MaAsLin2 analysis was performed to compare T48 and T72 to identify either families, genus, or ASV associated with specific conditions. No associations were found for any condition at T48, which was expected since the oils were added after the sampling. At T72 (24 h post oil addition), the C1000 and T1000 conditions revealed two associations at the family level. A positive association was found for the *Erysipelotrichaceae* for the C1000 condition. The other association found was a negative association for *Prevotellaceae* in the T1000 sample. It is noteworthy to mention that a positive trend was observed for the *Lactobacillaceae* in the T1000 sample compared to the negative control (P = 0.08).

## Discussion

In this study, a bioreactor system that could maintain a microbiota – derived from the piglet intestinal microbiota – in a continuous feeding system for a short time was successfully developed and its microbiota was modulated by the use of thymol. The system used piglet feed digested *in vitro* as the culture media to simulate the gut content of a pig. The IViDiS model generating the digested feed is a dynamic *in vitro* digestion system which attempts to mimic appropriately the digestion process occurring in piglets by simulating the ingestion pattern, destructuration of feed, enzymatic activities of the various digestion organs, pH profiles, and flow rates, including gastric emptying rate, according to data obtained from the literature and *in vivo* data. One limit of this experiment is that the exact composition (amino acid content, exact sugar content, exact lipid content, etc.) of the digestate in the different bags was not reanalyzed.

In studies simulating human digestion, an international consensus (INFOGEST) has emerged and a way to standardize *in vitro* digestion STATIC systems [27, 28]. For example, these guidelines allow for the standardization of not only the environmental conditions and the method itself but also on the type and source of chemicals to be used. Such international consensus were based on information gathered, partially from *in vivo* pig experiments, to simulate human digestion, since pig is recognized as a suitable *in vivo* model [28]. These methods cannot be applied systematically to a piglet system that is using a DYNAMIC model. The INFOGEST method, if applied, could not provide sufficient amount of digesta to feed, in continuum, for more than 72 h, the bioreactors. The IViDiS model is an entirely adjustable model that can adapt its digestion profiles to simulate any monogastric animal, as long as *in vivo* data is available. Chemical composition of each digestive solution is well controlled and developed from *in vivo* data available (human, pig or piglet, in this study), resulting in solutions which may be similar to other dynamic in vitro models available, such as the TIM, HGS or the DGM models, used for simulating human conditions [40, 41].

The microbiota of the bioreactor was firstly characterized according to incubation time. When looking at the alpha diversity, the three diversity indexes at T48 were lower compared to T0 but were more stable between T48 and T72. This result was in accordance with other studies that reported a loss of alpha diversity between 6 and 24 h in the bioreactor [18, 42]. This finding could be explained by the difficulty to dissociate the viable from the nonviable bacteria within the microbiota [43], which makes it hard to know which bacterial species in the bioreactor’s inoculum are actually viable before the start of each experiment and therefore possibly cultivable in the bioreactor. Since T0 was closely related to the inoculum sample, improving the preparation of the inoculum (for example by reducing oxygen contact with the sample or reducing the time between sample collection and freezing) could increase the diversity and the viability of some bacterial population, therefore increasing the number of families that could grow *in vitro*. However, this loss of diversity seems unavoidable, as even in an experiment where the inoculum was prepared in an anaerobic cabinet, such a loss was observed [44]. This loss of diversity could also be due to the culture conditions themselves. For example, plausible cause for the loss of *Methanobacteriaceae* could be the atmosphere composition. In fact, in the system, gaseous nitrogen was added to create an anaerobic environment. However, *Methanobacteriaceae* requires the reduction of CO_2_ with H_2_ to produce energy [45]. Replacing the gas mixture with a mix of these gases could help mitigate this problem in future studies. Another possibility for the improvement of the system would be to use redox probes to monitor the redox potential of the system and the analysis of the gaseous atmosphere of the reactor. This would allow to measure if the system could be left without N2 gaz administration as the anaerobic status of the system could be maintained by the microbiota activity [26]. Another possibility for the difference of composition between T0 and T72 was the absence of the host physiology. For example, *Spirochaetaceae* were not observed at T72 when they were clearly present at T0. Members in this family are host-associated, therefore not well suited to grow inside a bioreactor [46]. Some *in vitro* systems have included in their bioreactors mucin-coated beads to better simulate the host and give the microbiota an environment to attach itself [47]. This could be an improvement to be added to our system. On the other hand, the downside of using mucin beads is that they have to be replaced as the microbiota consumes the mucin. The difference in alpha diversity between the inoculum and the samples may also have come from the growth speed of certain bacterial populations. For example, genera in *Methanobacteriaceae* have highly variable growth time, varying between 3 and 40 h of minimal doubling time [45]. However perfectible this system might be, the bioreactor was more than able to maintain a complex and rich community of bacteria. Using the system allows to rapidly evaluate if an experimental condition modifies the establishment of the bioreactor microbiota and thus has a potential to be effective *in vivo*.

For the beta diversity, a significant variation of the microbiota composition in term of the relative abundance of different microbiota members was observed over time and when compared with the inoculum or piglet rectal swabs. This remains the major limitation with the use of bioreactors in general. Indeed, this variation was expected, since most studies performed in various types of bioreactors have reported a clear shift in microbiota composition over time [48, 49]. Since our experiment ended at 72 h, microbiota variations beyond 72 h are not known, but are likely to continue to shift. For example, the system developed by Tanner et al. was kept working for three days in batch mode and five days in continuous operation [23] for a total of eight stabilization days. Other systems, such as the system by McDonald et al., reported that 36 days of system run time was needed before reaching a state that was considered stable [50]. Our system was designed to be able to evaluate short term modifications, which enabled fast screening of feed additive and reduced the risk of system crash and this on a developing microbiota. Another possibility for the difference in beta diversity between the bioreactor and the inoculum comes from the culture media preparation. At the end of the culture media preparation, a crude filtration step was necessary to remove big undigested feed that could block the feeding tube, which is a limitation of the present system, a limitation not found when using lab-made culture media. This filtration probably reduced the culture media concentration of undigested complex sugars, such as starch or cellulose, reducing the growth ability of bacteria that relies on this carbon source, such as *Ruminococcaceae* [51]. Considering that the microbiota is still slowly evolving, the results amassed in this study could be considered useful to rapidly assess changes brought by a modification of the experimental conditions on a developing microbiota.

When comparing the microbiota, using replication of the experiment as a factor, a significant difference was found but was also expected. In animal studies, differences in microbiota composition between farms or batches of animals have been observed [52–54], even when an animal facility where controlled conditions are used [55]. Moreover, another study also found a higher reproducibility between reactors compared to between experiments, even if the fecal inoculum came from the same donor [50]. In our study, this variation in microbiota composition could come from the different batch of culture media used, which differed between experiments, which can be a negative side of not using lab-defined culture media.

As a proof of concept, the effect of essential oils on the bioreactor microbiota was investigated to test the possibility of microbiota manipulation in the bioreactor. Essential oils are used in pig feed to improve intestinal health [11, 56]. Essential oils used in the system were added into the culture media feeding bottles in order to gradually add the oils into the reactor, unlike another study where the oil was immediately added to the test bacteria [57]. Concentrations of 200 ppm and 1000 ppm were used for these two oils, based on the minimal inhibitory concentration (MIC) against *Salmonella* observed in the literature [58] and in previous work conducted in our lab [58]. These concentrations therefore respectively represent a sub-minimal and over-optimal concentration of oils. In the bioreactor, carvacrol and thymol, at either concentration, had no effect on the 16S copy number and Enterobacteria levels evaluated with qPCR. Moreover, almost no effect could be observed globally on the microbiota by 16S sequencing. Thymol at the concentration of 1000 ppm increased the levels of lactobacilli observed by qPCR. In the bioinformatics analysis of the sequencing results, a MaAsLin2 analysis was used to identify biomarkers associated with the oils at T72. No significant associations were found for either the *Lactobacillus* or the *Lactobacillaceae* family, however a positive trend was observed for the *Lactobacillaceae* in the T1000 sample compared to the negative control. Thymol used as a feed additive in broiler chickens has shown a positive effect on *Lactobacillus* [59]. These results potentially suggest a modest but specific effect of the oil on the intestinal microbiota, using *in vivo* models. In other studies, thymol has shown beneficial effects on pig intestinal health, such as a reduction of the diarrhea scores and an improvement of the jejunal barrier function, but no direct effect on the microbiota [60, 61].

Even though an increase of lactobacilli was found with qPCR, no effect of the oils was measured on the alpha and beta diversities. This result was unexpected, since minimal inhibitory (MIC) concentrations for thymol and carvacrol varies from 200 to 700 ppm for some bacterial species [56]. On farms, the absence or the negligible effect of thymol on the alpha and beta diversity of the microbiota has also been observed in weaned piglets, where the addition of 500 ppm of thymol in the feed had no impact on the microbiota composition [62].

Only two bacterial populations were associated with the use of the oils. A negative association was found for the *Prevotellaceae* for the T1000 condition and a positive association for the *Erysipelotrichaceae* was found for the C1000 condition compared to the negative control. In control conditions, an increase of the relative abundance of *Prevotellaceae* was gradually observed between T0 to T72. The negative impact of thymol addition corresponded to the return of *Prevotellaceae* at the proportion measured in the T0 samples. Growth over time of *Prevotellaceae* was also observed in another bioreactor system over a period of seven days [23]. Members of the *Prevotellaceae* family are found to be both beneficial and not for the animals in the literature. Some studies have shown *Prevotellaceae* improves health by improving glucose metabolism, while others report it could be involved in opportunistic infections or even inflammatory bowel disease [63–65].

## Conclusions

A short-term *in vitro* bioreactor assay was developed to maintain a complex microbiota derived from the piglet colonic microbiota with the use of an *in-vitro* digestate from piglet feed as the culture media. This system allowed fast screening of feed additive before their *in vivo* evaluation. This study, by using essential oils, confirmed that the bioreactor’s microbiota can be manipulated by increasing lactobacilli levels using a high concentration of thymol. This system could therefore become a useful screening tool and a complementary first step to classic *in vitro* bacteriology that would lead to the selections of the best experimental conditions to be applied *in vivo*. The system rapidly and safely identifies options that are the most likely to impact the animal’s microbiota therefore that have a high chance of being able to modify the gut microbiota in following *in vivo* trials. This would result in a reduction of the number of experimental groups needed for hypothesis testing *in vivo*.

## Additional files

Below is the link to the electronic supplementary material.

**Additional File 1:** Relative abundance of the microbiota composition at the phylum level for T0, T48, and T72

**Additional File 2:** Relative abundance of the microbiota composition at the family level for T0, T48, and T72

**Additional File 3:** Photo of the bioreactor setup. Major components of the system are highlighted and identified

**Additional File 4:** File containing all supplementary Tables 1 to 7, with their associated caption

## Data Availability

The datasets generated and analysed during the current study are available in the NCBI SRA database, with the BioProject ID PRJNA821632.

## Declarations

### Prior publication

Data have not been published previously.
