# Supplementary figures and images for: A short-term bioreactor assay to assess the effect of essential oils on a microbiota derived from piglet’s intestinal content
Source: Acta Vet Scand. 2023 May 19;65:17. doi: 10.1186/s13028-023-00679-w (PMC10199583; doi:10.1186/s13028-023-00679-w)

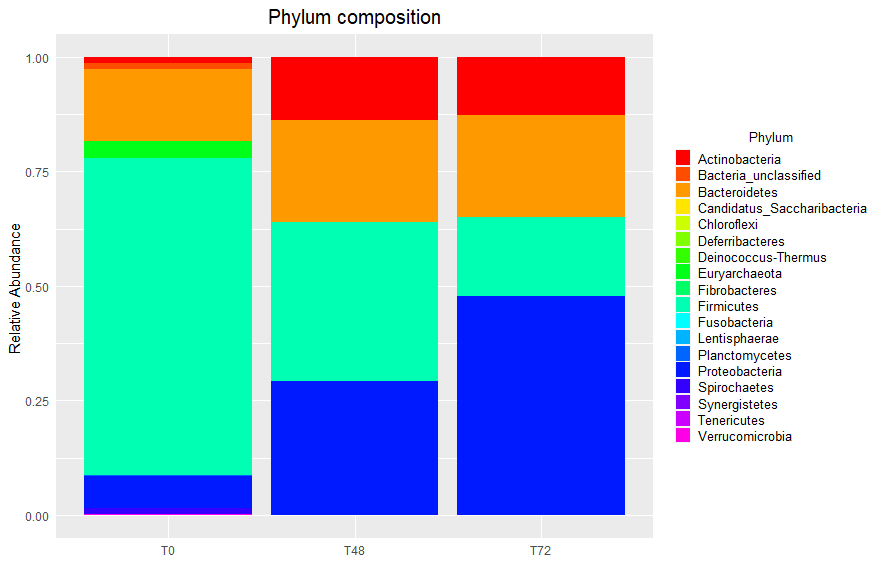

Supplement: Supplementary file 1 — Additional File 1: Relative abundance of the microbiota composition at the phylum level for T0, T48, and T72 [file 13028_2023_679_MOESM1_ESM.png]

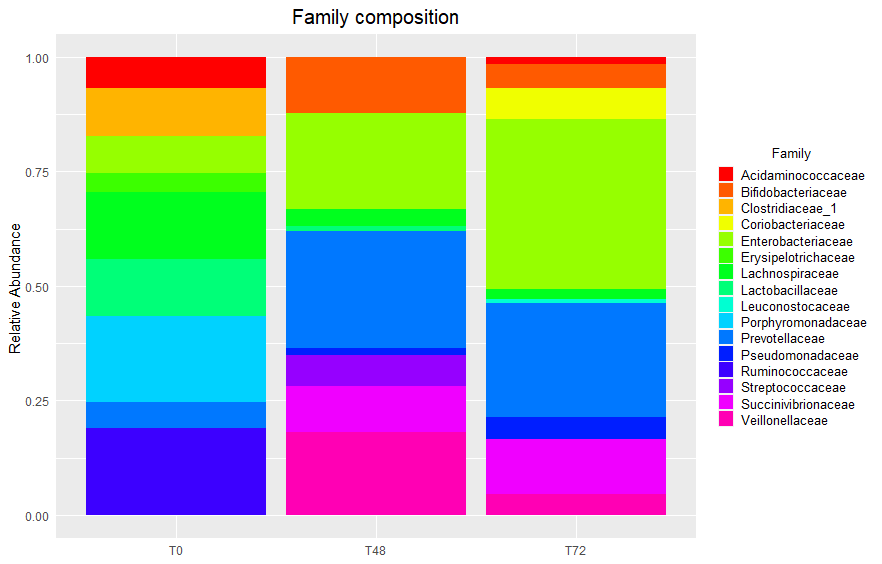

Supplement: Supplementary file 2 — Additional File 2: Relative abundance of the microbiota composition at the family level for T0, T48, and T72 [file 13028_2023_679_MOESM2_ESM.png]

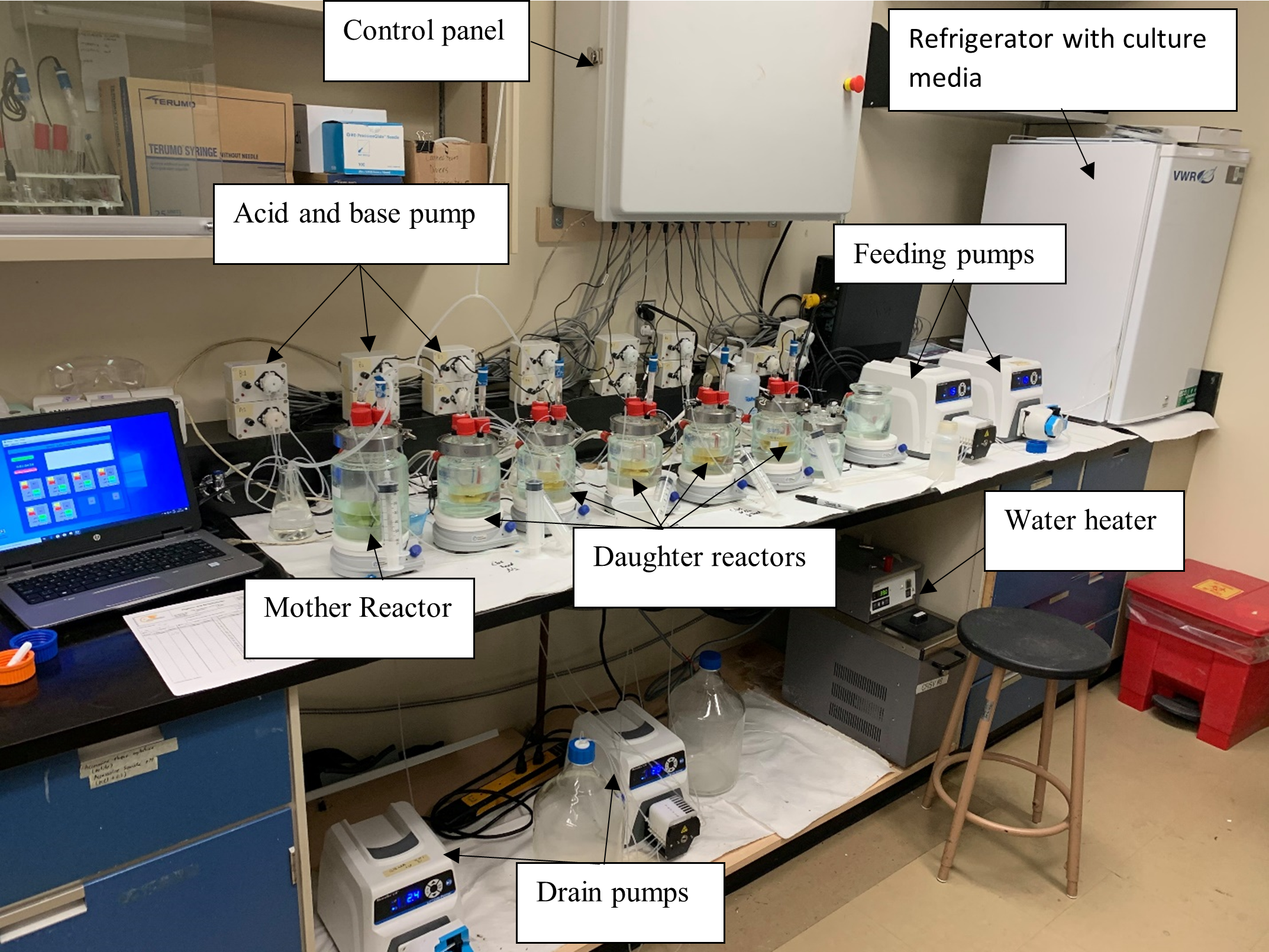

Supplement: Supplementary file 3 — Additional File 3: Photo of the bioreactor setup. Major components of the system are highlighted and identified [file 13028_2023_679_MOESM3_ESM.png]
