# Supplementary material for: A short-term bioreactor assay to assess the effect of essential oils on a microbiota derived from piglet’s intestinal content
Source: Acta Vet Scand. 2023 May 19;65:17. doi: 10.1186/s13028-023-00679-w (PMC10199583; doi:10.1186/s13028-023-00679-w)
Supplement: Supplementary file 4 — Additional File 4: File containing all supplementary Tables 1 to 7, with their associated caption [file 13028_2023_679_MOESM4_ESM.docx]

| Kingdom | Phylum | Time |
| --- | --- | --- |
| Bacteria | Candidatus_Saccharibacteria | T0 |
| Bacteria | Deferribacteres | T0 |
| Archaea | Euryarchaeota | T0 |
| Bacteria | Fibrobacteres | T0 |
| Bacteria | Lentisphaerae | T0 |
| Bacteria | Planctomycetes | T0 |
| Bacteria | Spirochaetes | T0 |
| Bacteria | Synergistetes | T0 |
| Bacteria | Tenericutes | T0 |
| Bacteria | Chloroflexi | T72 |
| Bacteria | Fusobacteria | T72 |

Supplementary table 1: List of unique Phylum present either at T0 or T72, based on Venn diagram.

| Phylum | Family | Number of Sequences | Percentage of all T0 sequences |
| --- | --- | --- | --- |
| Euryarchaeota | Methanobacteriaceae | 4152 | 3.600 |
| Spirochaetes | Spirochaetaceae | 1435 | 1.200 |
| Proteobacteria | Desulfovibrionaceae | 694 | 0.600 |
| Bacteroidetes | Bacteroidetes_unclassified | 288 | 0.200 |
| Verrucomicrobia | Subdivision5_unclassified | 138 | 0.100 |
| Planctomycetes | Planctomycetaceae | 67 | 0.060 |
| Spirochaetes | Spirochaetales_unclassified | 66 | 0.060 |
| Planctomycetes | Planctomycetes_unclassified | 58 | 0.050 |
| Lentisphaerae | Oligosphaeraceae | 51 | 0.040 |
| Proteobacteria | Oxalobacteraceae | 36 | 0.030 |
| Euryarchaeota | Methanomassiliicoccaceae | 24 | 0.020 |
| Proteobacteria | Campylobacteraceae | 17 | 0.010 |
| Synergistetes | Synergistaceae | 16 | 0.010 |
| Lentisphaerae | Victivallaceae | 15 | 0.010 |
| Bacteroidetes | Marinilabiliaceae | 13 | 0.010 |
| Firmicutes | Syntrophomonadaceae | 11 | 0.009 |
| Verrucomicrobia | Verrucomicrobiaceae | 11 | 0.009 |
| Tenericutes | Anaeroplasmataceae | 10 | 0.009 |
| Fibrobacteres | Fibrobacteraceae | 9 | 0.007 |
| Proteobacteria | Betaproteobacteria_unclassified | 9 | 0.008 |
| Firmicutes | Planococcaceae | 6 | 0.005 |
| Bacteroidetes | Rikenellaceae | 4 | 0.003 |
| Candidatus_Saccharibacteria | Candidatus_Saccharibacteria_unclassified | 4 | 0.003 |
| Proteobacteria | Pasteurellaceae | 4 | 0.003 |
| Actinobacteria | Corynebacteriaceae | 3 | 0.003 |
| Deferribacteres | Deferribacteraceae | 2 | 0.001 |
| Firmicutes | Bacillaceae_1 | 1 | 0.001 |
| Firmicutes | Bacillaceae_2 | 1 | 0.001 |
| Firmicutes | Catabacteriaceae | 1 | 0.001 |
| Proteobacteria | Desulfovibrionales_unclassified | 1 | 0.001 |
| Verrucomicrobia | Puniceicoccaceae | 1 | 0.001 |

Supplementary table 2: List of unique Families present at T0, with their associated Phylum, number of sequences and the percentage of total sequences at T0.

| Phylum | Family | Number of Sequences | Percentage of all T72 sequences |
| --- | --- | --- | --- |
| Actinobacteria | Actinobacteria_unclassified | 1 | 0.0003 |
| Actinobacteria | Dietziaceae | 3 | 0.0009 |
| Actinobacteria | Streptomycetaceae | 1 | 0.0003 |
| Bacteroidetes | Cytophagaceae | 2 | 0.0006 |
| Bacteroidetes | Flavobacteriaceae | 1 | 0.0003 |
| Chloroflexi | Sphaerobacteraceae | 2 | 0.0006 |
| Firmicutes | Bacillales_Incertae_Sedis_XI | 1 | 0.0003 |
| Firmicutes | Carnobacteriaceae | 4 | 0.0010 |
| Firmicutes | Selenomonadales_unclassified | 2 | 0.0006 |
| Fusobacteria | Fusobacteriaceae | 15 | 0.0040 |
| Proteobacteria | Aeromonadales_unclassified | 1 | 0.0003 |
| Proteobacteria | Alphaproteobacteria_unclassified | 1 | 0.0003 |
| Proteobacteria | Bradyrhizobiaceae | 3 | 0.0009 |
| Proteobacteria | Burkholderiaceae | 1 | 0.0003 |
| Proteobacteria | Burkholderiales_unclassified | 1 | 0.0003 |
| Proteobacteria | Rhizobiales_unclassified | 1 | 0.0003 |
| Proteobacteria | Sphingomonadaceae | 3 | 0.0009 |
| Verrucomicrobia | Subdivision3_unclassified | 1 | 0.0003 |

Supplementary table 3: List of unique Families present at T72, with their associated Phylum, number of sequences and the percentage of total sequences at T72.

| Phylum | Time | Association |
| --- | --- | --- |
| Bacteria_unclassified | T48 | -2.88 |
| Lentisphaerae | T48 | -0.85 |
| Planctomycetes | T48 | -0.82 |
| Verrucomicrobia | T48 | -0.56 |
| Synergistetes | T48 | -0.56 |
| Spirochaetes | T48 | -0.40 |
| Archaea.Euryarchaeota | T48 | -0.39 |
| Firmicutes | T48 | -0.33 |
| Proteobacteria | T48 | 0.55 |
| Actinobacteria | T48 | 0.90 |
| Bacteria_unclassified | T72 | -2.86 |
| Lentisphaerae | T72 | -0.85 |
| Planctomycetes | T72 | -0.82 |
| Firmicutes | T72 | -0.60 |
| Verrucomicrobia | T72 | -0.56 |
| Synergistetes | T72 | -0.56 |
| Spirochaetes | T72 | -0.40 |
| Archaea.Euryarchaeota | T72 | -0.39 |
| Proteobacteria | T72 | 0.81 |
| Actinobacteria | T72 | 0.96 |

Supplementary table 4: MaAsLin association at the Phylum level for T0, T48 and T72, using time as the factor.

| Phylum | Family | Time | Association |
| --- | --- | --- | --- |
| Firmicutes | Ruminococcaceae | T48 | -3.03 |
| Bacteroidetes | Porphyromonadaceae | T48 | -2.98 |
| Firmicutes | Firmicutes_unclassified | T48 | -2.91 |
| Bacteria_unclassified | Bacteria_unclassified | T48 | -2.87 |
| Firmicutes | Clostridiales_unclassified | T48 | -2.82 |
| Firmicutes | Clostridia_unclassified | T48 | -2.65 |
| Firmicutes | Peptostreptococcaceae | T48 | -2.47 |
| Bacteroidetes | Bacteroidales_unclassified | T48 | -2.46 |
| Firmicutes | Erysipelotrichaceae | T48 | -2.44 |
| Firmicutes | Peptococcaceae_1 | T48 | -2.19 |
| Firmicutes | Eubacteriaceae | T48 | -2.15 |
| Proteobacteria | Desulfovibrionaceae | T48 | -2.10 |
| Firmicutes | Clostridiaceae_1 | T48 | -1.91 |
| Proteobacteria | Proteobacteria_unclassified | T48 | -1.64 |
| Planctomycetes | Planctomycetes_unclassified | T48 | -1.18 |
| Firmicutes | Lachnospiraceae | T48 | -1.07 |
| Lentisphaerae | Oligosphaeraceae | T48 | -0.99 |
| Firmicutes | Enterococcaceae | T48 | -0.84 |
| Bacteroidetes | Bacteroidaceae | T48 | -0.81 |
| Firmicutes | Lactobacillaceae | T48 | -0.71 |
| Euryarchaeota | Methanomassiliicoccaceae | T48 | -0.65 |
| Proteobacteria | Betaproteobacteria_unclassified | T48 | -0.63 |
| Proteobacteria | Oxalobacteraceae | T48 | -0.61 |
| Synergistetes | Synergistaceae | T48 | -0.60 |
| Verrucomicrobia | Subdivision5_unclassified | T48 | -0.59 |
| Proteobacteria | Campylobacteraceae | T48 | -0.50 |
| Bacteroidetes | Bacteroidetes_unclassified | T48 | -0.50 |
| Spirochaetes | Spirochaetales_unclassified | T48 | -0.49 |
| Verrucomicrobia | Verrucomicrobiaceae | T48 | -0.46 |
| Planctomycetes | Planctomycetaceae | T48 | -0.45 |
| Firmicutes | Syntrophomonadaceae | T48 | -0.44 |
| Spirochaetes | Spirochaetaceae | T48 | -0.42 |
| Euryarchaeota | Methanobacteriaceae | T48 | -0.38 |
| Bacteroidetes | Rikenellaceae | T48 | -0.35 |
| Tenericutes | Anaeroplasmataceae | T48 | -0.34 |
| Fibrobacteres | Fibrobacteraceae | T48 | -0.33 |
| Bacteroidetes | Marinilabiliaceae | T48 | -0.32 |
| Lentisphaerae | Victivallaceae | T48 | -0.27 |
| Candidatus_Saccharibacteria | Candidatus_Saccharibacteria_unclassified | T48 | -0.26 |
| Proteobacteria | Enterobacteriaceae | T48 | 0.41 |
| Bacteroidetes | Prevotellaceae | T48 | 0.65 |
| Firmicutes | Streptococcaceae | T48 | 1.46 |
| Firmicutes | Veillonellaceae | T48 | 1.54 |
| Proteobacteria | Pseudomonadaceae | T48 | 1.79 |
| Actinobacteria | Bifidobacteriaceae | T48 | 2.89 |
| Firmicutes | Firmicutes_unclassified | T72 | -2.95 |
| Bacteria_unclassified | Bacteria_unclassified | T72 | -2.77 |
| Firmicutes | Clostridia_unclassified | T72 | -2.60 |
| Bacteroidetes | Porphyromonadaceae | T72 | -2.55 |
| Firmicutes | Ruminococcaceae | T72 | -2.52 |
| Firmicutes | Peptostreptococcaceae | T72 | -2.52 |
| Firmicutes | Clostridiales_unclassified | T72 | -2.43 |
| Bacteroidetes | Bacteroidales_unclassified | T72 | -2.21 |
| Proteobacteria | Desulfovibrionaceae | T72 | -2.15 |
| Firmicutes | Clostridiaceae_1 | T72 | -1.93 |
| Firmicutes | Peptococcaceae_1 | T72 | -1.92 |
| Firmicutes | Lactobacillaceae | T72 | -1.84 |
| Firmicutes | Eubacteriaceae | T72 | -1.67 |
| Proteobacteria | Proteobacteria_unclassified | T72 | -1.49 |
| Firmicutes | Enterococcaceae | T72 | -1.23 |
| Planctomycetes | Planctomycetes_unclassified | T72 | -1.18 |
| Firmicutes | Erysipelotrichaceae | T72 | -1.13 |
| Lentisphaerae | Oligosphaeraceae | T72 | -0.99 |
| Firmicutes | Lachnospiraceae | T72 | -0.96 |
| Euryarchaeota | Methanomassiliicoccaceae | T72 | -0.65 |
| Proteobacteria | Betaproteobacteria_unclassified | T72 | -0.63 |
| Proteobacteria | Oxalobacteraceae | T72 | -0.61 |
| Synergistetes | Synergistaceae | T72 | -0.60 |
| Verrucomicrobia | Subdivision5_unclassified | T72 | -0.59 |
| Proteobacteria | Campylobacteraceae | T72 | -0.50 |
| Bacteroidetes | Bacteroidetes_unclassified | T72 | -0.50 |
| Spirochaetes | Spirochaetales_unclassified | T72 | -0.49 |
| Verrucomicrobia | Verrucomicrobiaceae | T72 | -0.46 |
| Planctomycetes | Planctomycetaceae | T72 | -0.45 |
| Firmicutes | Syntrophomonadaceae | T72 | -0.44 |
| Spirochaetes | Spirochaetaceae | T72 | -0.42 |
| Bacteroidetes | Rikenellaceae | T72 | -0.40 |
| Euryarchaeota | Methanobacteriaceae | T72 | -0.38 |
| Tenericutes | Anaeroplasmataceae | T72 | -0.34 |
| Fibrobacteres | Fibrobacteraceae | T72 | -0.33 |
| Firmicutes | Clostridiales_Incertae_Sedis_XIII | T72 | -0.33 |
| Bacteroidetes | Marinilabiliaceae | T72 | -0.32 |
| Lentisphaerae | Victivallaceae | T72 | -0.27 |
| Candidatus_Saccharibacteria | Candidatus_Saccharibacteria_unclassified | T72 | -0.25 |
| Proteobacteria | Gammaproteobacteria_unclassified | T72 | 0.33 |
| Actinobacteria | Coriobacteriaceae | T72 | 0.55 |
| Proteobacteria | Enterobacteriaceae | T72 | 0.68 |
| Bacteroidetes | Prevotellaceae | T72 | 0.71 |
| Firmicutes | Veillonellaceae | T72 | 1.11 |
| Proteobacteria | Moraxellaceae | T72 | 1.15 |
| Firmicutes | Leuconostocaceae | T72 | 1.16 |
| Proteobacteria | Pseudomonadaceae | T72 | 2.63 |
| Actinobacteria | Bifidobacteriaceae | T72 | 2.74 |

Supplementary table 5: MaAsLin association at the Family level for T0, T48 and T72, using time as the factor.

| **Factor** | **Phylum** | **Family** | **Number of sequences** | **Percentage of all factor-associated sequences** |
| --- | --- | --- | --- | --- |
| T72 | Chloroflexi | Sphaerobacteraceae | 2 | 0.0006 |
| T72 | Bacteroidetes | Cytophagaceae | 2 | 0.0006 |
| T0 | Firmicutes | Planococcaceae | 6 | 0.0052 |
| Piglet | Firmicutes | Clostridiales_Incertae_Sedis_XI | 166 | 0.0522 |
| Piglet | Actinobacteria | Actinomycetaceae | 142 | 0.0447 |
| Piglet | Proteobacteria | Alphaproteobacteria_unclassified | 89 | 0.0280 |
| Piglet | Proteobacteria | Helicobacteraceae | 65 | 0.0205 |
| Piglet | Actinobacteria | Dermabacteraceae | 44 | 0.0138 |
| Piglet | Proteobacteria | Caulobacteraceae | 28 | 0.0088 |
| Piglet | Proteobacteria | Desulfovibrionales_unclassified | 24 | 0.0076 |
| Piglet | Firmicutes | Peptoniphilaceae | 24 | 0.0076 |
| Piglet | Proteobacteria | Comamonadaceae | 21 | 0.0066 |
| Piglet | Chlamydiae | Chlamydiaceae | 19 | 0.0060 |
| Piglet | Proteobacteria | Burkholderiaceae | 8 | 0.0025 |
| Piglet | Actinobacteria | Brevibacteriaceae | 8 | 0.0025 |
| Piglet | Proteobacteria | Halomonadaceae | 8 | 0.0025 |
| Piglet | Firmicutes | Bacillaceae_1 | 3 | 0.0009 |
| Piglet | Proteobacteria | Xanthomonadaceae | 3 | 0.0009 |
| Piglet | Proteobacteria | Neisseriaceae | 3 | 0.0009 |
| Piglet | Fusobacteria | Leptotrichiaceae | 3 | 0.0009 |
| Piglet | Firmicutes | Christensenellaceae | 3 | 0.0009 |
| Piglet | Bacteroidetes | Sphingobacteriaceae | 2 | 0.0006 |
| Piglet | Proteobacteria | Rhizobiales_unclassified | 2 | 0.0006 |
| Piglet | Proteobacteria | Shewanellaceae | 2 | 0.0006 |
| Piglet | Proteobacteria | Rhizobiaceae | 2 | 0.0006 |
| Piglet | Actinobacteria | Actinobacteria_unclassified | 2 | 0.0006 |

Supplementary table 6: Unique Families associated with either T72, T0 or piglet factor.

| **Factor** | **Genus** | **Numbers of sequences** | **Percentage of all factor-associated sequences** |
| --- | --- | --- | --- |
| T72 | Proteus | 173 | 0.0503 |
| T72 | Brochothrix | 6 | 0.0017 |
| T72 | Pseudomonadaceae_unclassified | 6 | 0.0017 |
| T72 | Listeria | 3 | 0.0009 |
| T72 | Bradyrhizobium | 3 | 0.0009 |
| T72 | Sphaerobacteraceae_unclassified | 2 | 0.0006 |
| T72 | Hymenobacter | 2 | 0.0006 |
| T0 | Lysinibacillus | 6 | 0.0052 |
| T0 | Synergistes | 4 | 0.0034 |
| T0 | Cellulosilyticum | 2 | 0.0017 |
| T0 | Lactobacillaceae_unclassified | 2 | 0.0017 |
| T0 | Robinsoniella | 2 | 0.0017 |
| Piglet | Dialister | 4057 | 1.2768 |
| Piglet | Catenibacterium | 1829 | 0.5756 |
| Piglet | Methanosphaera | 900 | 0.2833 |
| Piglet | Oribacterium | 552 | 0.1737 |
| Piglet | Mobiluncus | 115 | 0.0362 |
| Piglet | Ezakiella | 91 | 0.0286 |
| Piglet | Alphaproteobacteria_unclassified | 89 | 0.0280 |
| Piglet | Anaerococcus | 71 | 0.0223 |
| Piglet | Helicobacter | 65 | 0.0205 |
| Piglet | Veillonella | 50 | 0.0157 |
| Piglet | Brachybacterium | 44 | 0.0138 |
| Piglet | Pasteurella | 36 | 0.0113 |
| Piglet | Odoribacter | 29 | 0.0091 |
| Piglet | Caulobacter | 28 | 0.0088 |
| Piglet | Desulfovibrionales_unclassified | 24 | 0.0076 |
| Piglet | Peptoniphilus | 24 | 0.0076 |
| Piglet | Porphyromonas | 22 | 0.0069 |
| Piglet | Actinomyces | 20 | 0.0063 |
| Piglet | Acidovorax | 19 | 0.0060 |
| Piglet | Chlamydia | 19 | 0.0060 |
| Piglet | Yaniella | 17 | 0.0054 |
| Piglet | Salinicoccus | 16 | 0.0050 |
| Piglet | Megamonas | 16 | 0.0050 |
| Piglet | Jeotgalicoccus | 15 | 0.0047 |
| Piglet | Lactococcus | 9 | 0.0028 |
| Piglet | Brevibacterium | 8 | 0.0025 |
| Piglet | Halomonas | 8 | 0.0025 |
| Piglet | Ralstonia | 7 | 0.0022 |
| Piglet | Desulfovibrionaceae_unclassified | 7 | 0.0022 |
| Piglet | Actinobacillus | 7 | 0.0022 |
| Piglet | Rothia | 6 | 0.0019 |
| Piglet | Bradyrhizobiaceae_unclassified | 6 | 0.0019 |
| Piglet | Butyrivibrio | 4 | 0.0013 |
| Piglet | Actinomycetaceae_unclassified | 4 | 0.0013 |
| Piglet | Ruminobacter | 4 | 0.0013 |
| Piglet | Enhydrobacter | 3 | 0.0009 |
| Piglet | Trueperella | 3 | 0.0009 |
| Piglet | Kocuria | 3 | 0.0009 |
| Piglet | Neisseria | 3 | 0.0009 |
| Piglet | Leptotrichia | 3 | 0.0009 |
| Piglet | Christensenella | 3 | 0.0009 |
| Piglet | Peptostreptococcus | 3 | 0.0009 |
| Piglet | Acetivibrio | 3 | 0.0009 |
| Piglet | Sphingobacteriaceae_unclassified | 2 | 0.0006 |
| Piglet | Rhizobiales_unclassified | 2 | 0.0006 |
| Piglet | Stenotrophomonas | 2 | 0.0006 |
| Piglet | Moraxella | 2 | 0.0006 |
| Piglet | Bacillaceae_1_unclassified | 2 | 0.0006 |
| Piglet | Comamonas | 2 | 0.0006 |
| Piglet | Shewanella | 2 | 0.0006 |
| Piglet | Citrobacter | 2 | 0.0006 |
| Piglet | Kaistia | 2 | 0.0006 |
| Piglet | Actinobacteria_unclassified | 2 | 0.0006 |
| Piglet | Oxalobacter | 2 | 0.0006 |
| Piglet | Herminiimonas | 2 | 0.0006 |

**Supplementary table 7**: Unique Genus associated with either T72, T0 or piglet factor.
